# Effects of *Didymosphenia geminata* massive growth on stream communities: Smaller organisms and simplified food web structure

**DOI:** 10.1371/journal.pone.0193545

**Published:** 2018-03-01

**Authors:** Rubén Ladrera, Joan Gomà, Narcís Prat

**Affiliations:** Freshwater Ecology and Management (F.E.M.) Research Group, Department of Evolutionary Biology, Ecology and Environmental Sciences, Universitat de Barcelona, Barcelona, Catalonia, Spain; Argonne National Laboratory, UNITED STATES

## Abstract

This study aims to contribute to the understanding of the impact of *Didymosphenia geminata* massive growths upon river ecosystem communities’ composition and functioning. This is the first study to jointly consider the taxonomic composition and functional structure of diatom and macroinvertebrate assemblages in order to determine changes in community structure, and the food web alterations associated with this invasive alga. This study was carried out in the Lumbreras River (Ebro Basin, La Rioja, Northern Spain), which has been affected by a considerable massive growth of *D*. *geminata* since 2011. The study shows a profound alteration in both the river community composition and in the food web structure at the sites affected by the massive growth, which is primarily due to the alteration of the environmental conditions, thus demonstrating that *D*. *geminata* has an important role as an ecosystem engineer in the river. Thick filamentous mats impede the movement of large invertebrates—especially those that move and feed up on it—and favor small, opportunistic, herbivorous organisms, mainly chironomids, that are capable of moving between filaments and are aided by the absence of large trophic competitors and predators -prey release effect-. Only small predators, such as hydra, are capable of surviving in the new environment, as they are favored by the increase in chironomids, a source of food, and by the reduction in both their own predators and other midge predators -mesopredator release-. This change in the top-down control affects the diatom community, since chironomids may feed on large diatoms, increasing the proportion of small diatoms in the substrate. The survival of small and fast-growing pioneer diatoms is also favored by the mesh of filaments, which offers them a new habitat for colonization. Simultaneously, *D*. *geminata* causes a significant reduction in the number of diatoms with similar ecological requirements (those attached to the substrate). Overall, *D*. *geminata* creates a community dominated by small organisms that is clearly different from the existing communities in the same stream where there is an absence of massive growths.

## Introduction

The high transport capacity of our globalized society has allowed invasive species to become one of the main threats to biodiversity around the world, especially with relation to inland aquatic ecosystems [1,2]. The presence of invasive species in aquatic ecosystems is particularly disturbing within the Iberian Peninsula [3,4], where before 2010, 113 non-native species (including algae, fungi, mollusc, crustacean and fish taxa) had been intentionally or accidentally introduced [5]. Among the various invasive species described in Iberian rivers, we found the alga *Didymosphenia geminata*, which has been recently included in the Spanish Invasive Species Catalog (RD 630/2013).

*D*. *geminata* is a diatom that, under certain environmental conditions [6–10], is capable of producing a large amount of extracellular stalks, creating massive growths. These biological episodes can cover the river bed for several kilometers, profoundly altering the environmental river conditions. In line with this observation, many papers note that *D*. *geminata* has a considerable impact on aquatic ecosystems based on the assumption that the large biomass of this species will have negative consequences for other species [11].

We should therefore consider *D*. *geminata* an ecosystem engineer, which can be understood as an organism “that directly or indirectly controls the availability of resources to other organisms by causing physical state changes in biotic or abiotic materials” [12–14]. Habitat alteration has been highlighted as one of the main impacts that invasive species have on their host ecosystem’s structure and functionality [15]. However, studies focused on *D*. *geminata*’s effects on river community structure and functioning are scarce [6,16] or they are usually limited to describing taxonomic composition changes, and do not focus on the ecosystem’s other descriptive variables such as the functional structure or trophic relations in the river. These variables could prove useful for our understanding of the relationship between the community alterations caused by different environmental pressures [17,18], such as the new environment created by *D*. *geminata*’s massive growths. The use of descriptive ecosystem variables enabled us to obtain general conclusions independent of the geographical area studied [19,20] and allowed us to establish general conclusions relating to the risks associated with *D*. *geminata* invasions, as well as the mechanisms underlying such invasions, which in turn may prove useful for controlling this species’ growth.

Until now, most studies of the effects of *D*. *geminata* on aquatic riverine communities have not gone beyond analyzing the changes to the macroinvertebrate taxonomic composition. These studies note important alterations to the invertebrate assemblage under massive growth conditions: usually the chironomids’ density increases and the EPT importance decreases [11,16,21,22], but few studies have focused on understanding the functional structure together with the taxonomic composition of this assemblage [22]. There is also a paucity of scientific analysis of *D*. *geminata*’s effects on other organisms, such as diatoms. Among the few studies focused on this assemblage, Gillis and Lavoie [23], and Sanmiguel *et al*. [24], have recently shown the alterations caused by massive *D*. *geminata* growths on other algae, and, surprisingly, they discovered a higher level of diatom diversity. In both studies, the authors recognize the lack of strong and clear conclusions about both the causes and effects of *D*. *geminata* on diatom composition, and call for new studies to elucidate the role of *D*. *geminata* in the changes produced in biofilms growing on river hard substrates. In response to this call, we believe that studying the effects on the community using an approach that takes into account both functional traits and trophic relations will help to explain the mechanisms of *D*. *geminata* invasions and how they affect river community and host ecosystem.

Macroinvertebrates and diatoms are the assemblages that are most widely used as biological indicators of the condition of aquatic ecosystems [25–28], so new studies that aim to understand the role of *D*. *geminata* in the biomonitoring of river ecosystem conditions must take these assemblages into consideration.

In our previous studies, undertaken in the Lumbreras River (Ebro Basin, Northern Spain) we reported the first massive growth of *D*. *geminata* in the southern tributaries of the Ebro catchment [29], and investigated its wide distribution in the Iregua and Najerilla Basins [30]. We also established a link between massive growths in this Mediterranean area and hydrological regulation, high light intensity and the water’s low phosphate content [10]. Additionally, we determined an important impact of massive *D*. *geminata* growth on the macroinvertebrate assemblage [22]. The aim of this study is to better understand the impact of *D*. *geminata* on the whole river community, and to understand the changes to the functional characteristics of the algal and macroinvertebrate assemblages that are produced by its massive growths. The specific objectives of this work are: i) to assess the degree of change of the taxonomic composition and functional structure of diatom and macroinvertebrate assemblages related to massive growths of *D*. *geminata*; and ii) to determine if these changes led to large-scale trophic and community structure alterations in the river food webs.

Our working hypotheses are: i) the presence of large biomasses of *D*. *geminata* will result in a decrease in the number of organisms adapted to move or feed on the substrate and those fixed to it, be they invertebrates or diatoms; ii) smaller herbivores will be favored by the absence of competitors and predators due to the fact that large invertebrates cannot move between the filaments; and iii) the degree of community alteration and changes in trophic relations will be directly related to the biomass of the *D*. *geminata*.

## Methodology

### Study area

**Fig 1:**
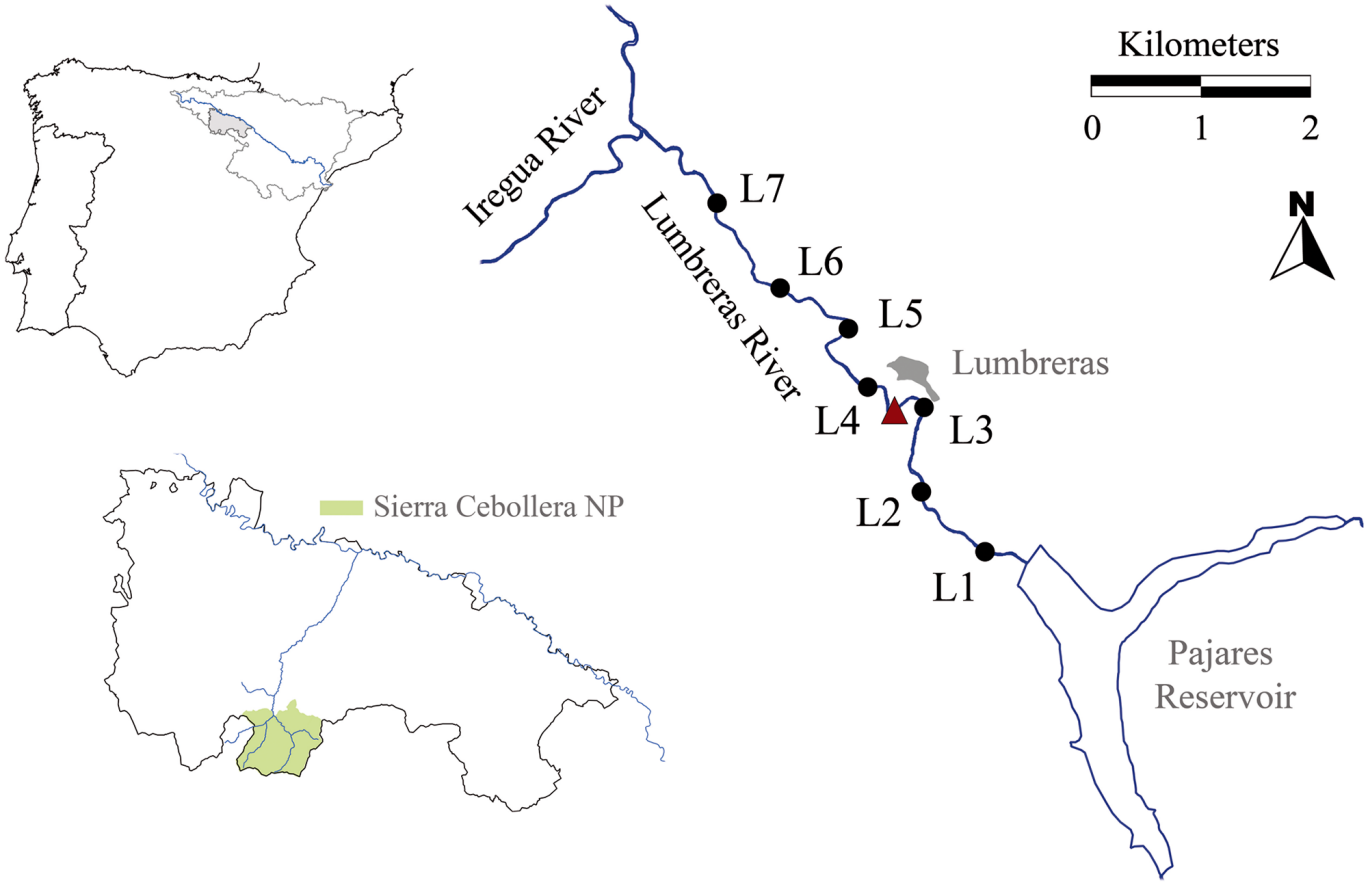
Map of the study area. Sampling sites are represented by black circles, and the red triangle shows the location of the Lumbreras Village’s sewage discharge. Green is used to show the “Sierra Cebollera” Natural Park, which includes the area studied.

The Lumbreras River is a mountain headwater river (average discharge 1.82 m^3^/s) located in the Sierra Cebollera Natural Park (La Rioja, northern Spain), within the Ebro Basin (Fig 1). It is the principal tributary of the Iregua River in the upper stretch of its catchment and it is regulated by the Pajares Reservoir (35 hm^3^), which clearly alters the river’s natural hydrograph (see Ladrera and Prat [29] for further details about the Lumbreras River’s hydrological regimen in relation to the Pajares Reservoir). Seven sites located downstream of the Pajares Reservoir were studied (L1-L7; 42°05’39”-42°07’07”N, 02°36’87”-02°38’37”O), the three closest to the dam (L1, L2 and L3) being heavily affected by the massive growth of *D*. *geminata* in summer, while there was no conspicuous growth in the other four (L4, L5, L6 and L7), located downstream of the Lumbreras Village’s sewage discharge. We consider massive growth to be when dense *D*. *geminata* mats with a thickness greater than 5 mm appear continuously along a river stretch longer than 1 km [10]. The first sampling site was located 400 m downstream of the Pajares Dam, and subsequent sites were located every 800 meters along the river’s course, all sites being within a stretch of 5 km. The selected sites had similar water discharge levels since there are no tributaries in the river section studied.

### Sampling strategy

**Fig 2:**
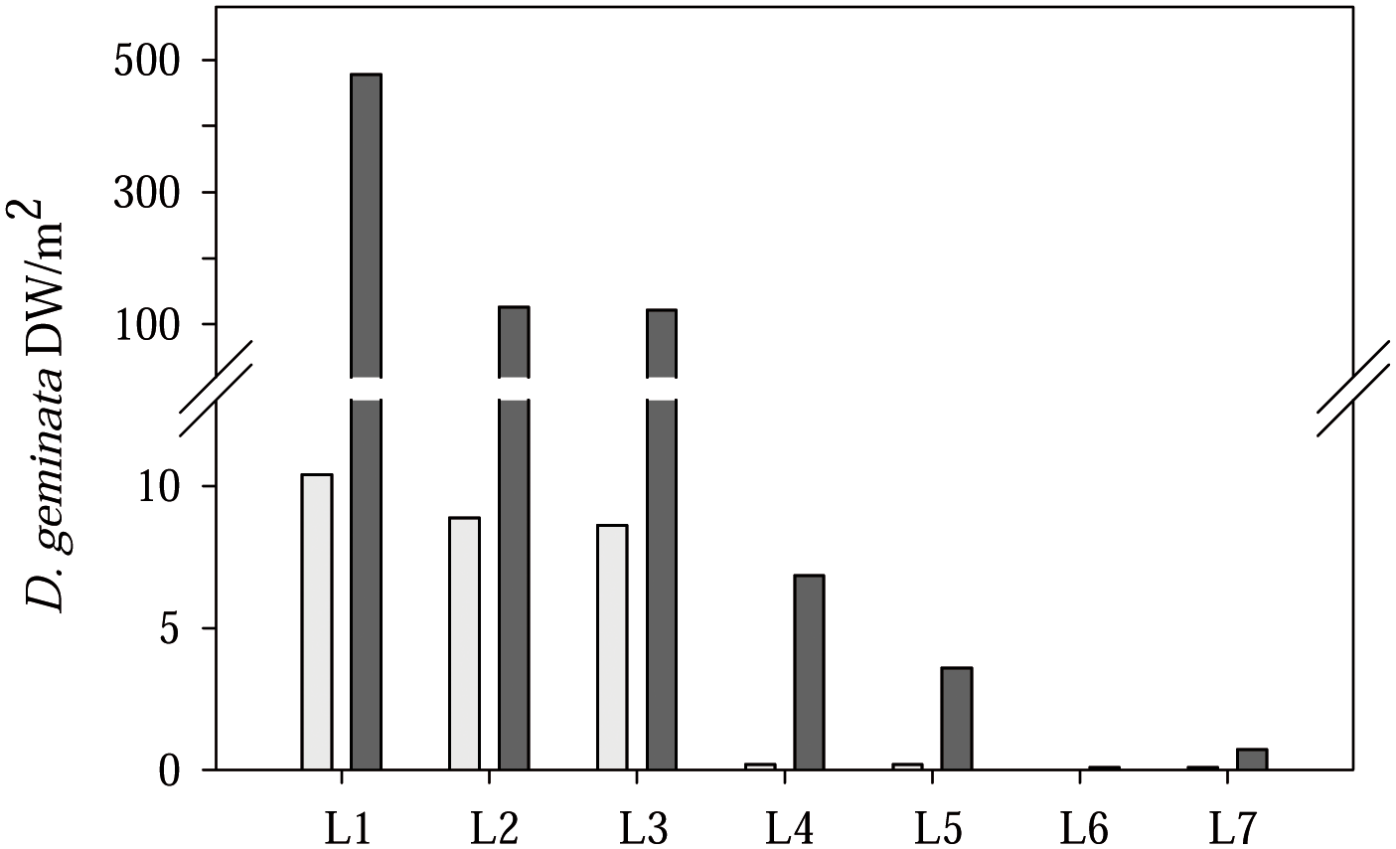
*D*. *geminata* biomass. *D*. *geminata* biomass (filaments gDW/m^2^) at every sample sites on June 22^nd^ and August 31^st^, when macroinvertebrate and diatom samples were collected.

Seven sampling sites, and two dates, June 22^nd^ and August 31^st^, were chosen for the study. We used two sampling periods in order to assess the effects of *D*. *geminata* growth on the river at different times. On June 22^nd^, the alga was in the early stages of growth, while on August 31^st^, it was fully grown. Sites L1, L2 and L3 showed *D*. *geminata* biomass of higher than 100 gDW/m^2^, being close to 500 gDW/m^2^ at site L1 (Fig 2). As stated previously, downstream of the Lumbreras discharge, the massive growth disappeared (due to higher phosphate levels, according to our previous studies [10] carried out in the Lumbreras River), although isolated mats of filaments were found at sites L4 and L5 on August 31^st^ (Fig 2). Consequently, the varying amounts of filaments of *D*. *geminata* present at the different sample sites allowed us to compare the diatom and macroinvertebrate assemblages in the river sections in order to understand the diverse impacts of this invasive alga.

### Environmental variables

For each site and sampling date (June 22^nd^ and August 31^st^, 2013) water temperature (°C), pH, conductivity (μS/cm) and dissolved oxygen levels (ppm) were measured *in situ*. Water was collected, filtered and kept frozen until the levels of Soluble Reactive Phosphorus (SRP) could be analysed in the laboratory following the acidic molybdate method [31] using a spectrophotometer (Shimadzu UV-1201). The quality of the riparian habitat was characterized using the QBR (Qualitat del Bosc de Ribera) Riparian Forest Quality Index [32], and the fluvial instream habitat was characterized using the IHF (Índice de Hábitat Fluvial) River Habitat Index [33], both indexes ranging from 0 to 100.

### Diatom sampling

At each site and on both dates, an area of 3–5 streambed cobbles larger than 10 cm and continuously covered by flowing water were brushed with a toothbrush (75 cm^2^ of each cobble) to collect diatoms into a 125 ml plastic jar, thus producing a single, pooled sample. Cobbles were randomly chosen at each site, and the area was measured with a plastic sample sheet. Samples were fixed in the field using 4% formaldehyde and taken to the laboratory to be identified. They were treated in order to obtain a clean frustule suspension via hydrogen peroxide (33%) oxidation, which was mounted in Naphrax. Using a ‘‘Polyvar” light microscope, at least 400 valves were counted to estimate the relative abundance of each taxon in the sample. The diatoms were identified at the lowest taxonomical level in line with the following authors’ methods: Krammer & Lange-Bertalot [34–38], Krammer [39], Lange-Bertalot and Krammer [40] and Reichardt [41].

In order to determine the biomass of the *D*. *geminata* samples, after sorting all other algal, plant, or invertebrate material, the stalks of the *D*. *geminata* were dried for 72 hours at 70°C for dry weight (DW) determination.

### Macroinvertebrate sampling

Multi-habitat samples for the analysis of the macroinvertebrate assemblage were collected using a 250 μm surber net in line with the MIQU sampling protocol (MacroInvertebrates QUantitative sampling protocol; for further details see Nuñez and Prat [42] and Ladrera and Prat [29]), covering every habitat present in the river. Samples were preserved in 4% formaldehyde and taken to the laboratory to be identified. The identification of macroinvertebrates was generally made to genus level, except for some Diptera subfamilies and Oligochaeta. Where necessary, sub-sampling was done in the sorting process, and at least 300 individuals per sample were counted.

### Community functional structure

Eight biological traits (Size class, Mobile, Pioneer, Adnate, Pedunculate, Pad, Stalk and Colonial) obtained from a published database based on species taxonomical levels [43] were used to describe the diatom assemblage’s functional structure. Size is categorized using 5 levels (with biovolume (in μm^3^) boundaries following a logarithmic evolution: 0 < class 1 < 100 ≤ class 2 < 300 ≤ class 3 < 600 ≤ class 4 < 1500 ≤class 5), while the other traits have values of 1 or 0, depending on whether each species possesses each trait or not.

For the macroinvertebrate assemblage, four biological traits (locomotion, substrate preferences, feeding habits and food) were considered, containing 35 categories obtained from Tachet *et al*. [44]. The traits in this database have an affinity score assigned for each taxa ranging from 0 to 5, from null affinity to high affinity, respectively [45]. The functional structure was calculated mostly based on genus level, always in line with the dataset requirements established by Tachet *et al*. [44]. To analyze the functional structure of both assemblages, a dataset of the relative abundance of traits per sample was built, for which the affinity of each taxon with each trait category was multiplied by the taxon’s abundance [45].

### Data analysis

Exponential regressions between *D*. *geminata* biomass (log transformed) and the abundance of diatom and macroinvertebrate taxa and biological traits were made in order to study their relations. To obtain more solid and representative values, only those taxa with a relative abundance higher than 1%, either for macroinvertebrates (grouped in families) as well as for diatoms (grouped by genus), were analyzed.

In order to establish the main links between environmental variables and diatom and macroinvertebrate assemblages, two DISTLM analyses were performed (PERMANOVA + for PRIMER [46]) based on species and genus abundance respectively. The diatom and macroinvertebrate distance matrices were created using the chord distance method after the assemblages’ data were ln (*x* + 1) transformed. The environmental variables were ln (*x* + 1) transformed and normalized. The DISTLM routine was based on the forward selection procedure and the AIC selection criteria [47], to obtain the environmental variables that accounted for further variation. For each assemblage, a dbRDA plot from the DISTLM analysis was used to visualize the final model. In each dbRDA plot, we show the environmental variables that were selected in the final model as obtained from the DISTLM analysis.

## Results

Every studied site showed on both sampling dates high dissolved oxygen concentration (values ranging from 8.67 to 10.06 ppmO_2_), low temperature (9.5–13.5°C) and conductivity (76.90–91.50 μS/cm) and slightly alkaline waters (pH ranged from 7.60 to 8.09). The only physicochemical variable which showed noticeable differences among sites was SRP, especially after Lumbreras sewage discharge, increasing from 0.012 ppm in L3 to 0.021 and 0.017 ppm in L4 in June and August respectively. QBR index ranged from 80 to 100 in every sites, except in L1 (QBR = 5), due to the removal of the riparian forest downstream of the Pajares Reservoir. IHF index increased from 53 to 72 along the longitudinal profile of Lumbreras River.

**Fig 3:**
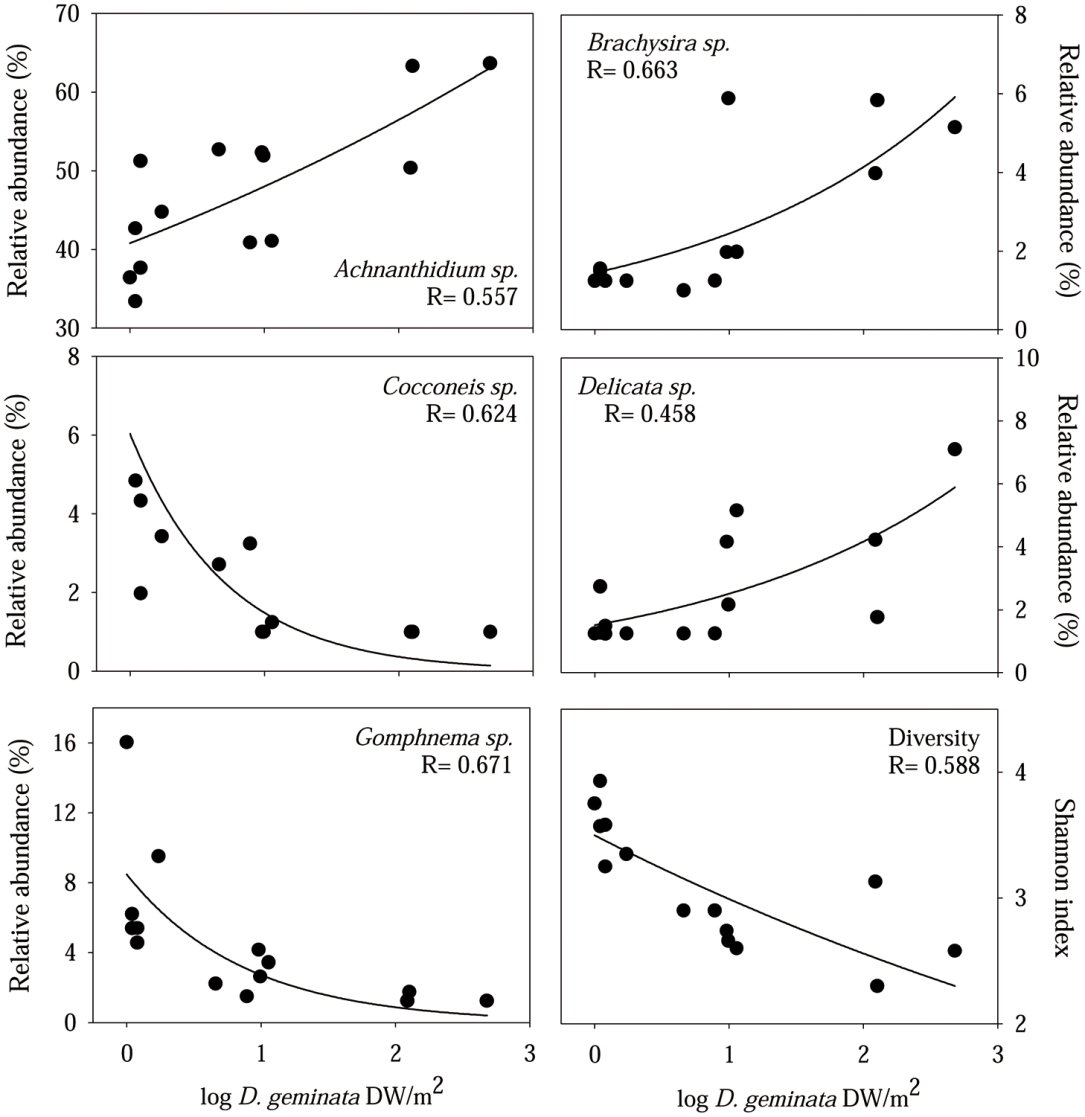
Relationship between *D*. *geminata* biomass and diatom genus and diversity. Exponential regressions between *D*. *geminata* biomass (log transformed) and diatom genus that were shown to be significant (p<0.05). (Lower right) Exponential regressions between *D*. *geminata* biomass (log transformed) and the Shannon diversity index of Diatom assemblages at each site, also proved to be statistically significant (p<0.05).

The relative abundance of *D*. *geminata* always remained below 3% of the total number of diatom cells even on sites affected by massive growths of this alga (L1, L2 and L3). The taxonomic composition of the diatom assemblage, excluding *D*. *geminata*, totaled 77 taxa, and was dominated by several species of *Achnanthidium* sp., mainly *Achnanthidium minutissimum*, which presents a relative abundance of higher than 20% in all samples. Diatom species were grouped by genus in order to achieve more consistent results in the regression analysis of the *D*. *geminata* biomass. 11 genera showed a relative abundance of higher than 1% at least in one site: *Achnanthes* spp. (including *A*. *atomus* Hustedt, *A*. *flexella* (Kützing) Brun and *A*. *pyrenaica* Hustedt), *Achnanthidium* spp. (containing *A*. *biasolettianum* (Grunow) Lange-Bertalot and *A*. *minutissimum* (Kützing) Czarnecki), *Brachysira* sp. (*B*. *neoexilis* Lange-Bertalot), *Cocconeis* sp. (with *C*. *placentula* Ehrenberg, and *C*. *placentula* Ehrenberg var. *euglypta* (Ehrenberg) Grunow), *Cyclotella* spp. (summing *C*. *radiosa* (Grunow) Lemmermann and *C*. *stelligera* Cleve & Grunow), *Cymbella* spp. (containing *C*. *amphicephala* Naegeli and *C*. *perparva* Krammer), *Delicata* sp. (summing *D*. *delicatula* (Kützing) Krammer var. *alpestris* Krammer, *D*. *delicatula* (Kützing) Krammer), *Encyonema* spp. (including *E*. *minutum* (Hilse) Mann and *E*. *silesiacum* (Bleisch) Mann), *Fragilaria* spp. (with *F*. *arcus* Ehrenberg, *F*. *brevistriata* Grunow, *F*. *capucina* Desmazieres, *F*. *capucina* Desmazieres var. *Vaucheriae* (Kützing) Lange-Bertalot, *F*. *elliptica* Schumann, *F*. *parasitica* (Smith) Grunow, *F*. *rumpens* (Kützing) Carlson, *F*. *tenera* (Smith) Lange-Bertalot, *F*. *ulna* (Nitzsch.) Lange-Bertalot, *F*. *virescens* Ralfs and *Fragilaria* sp.), *Gomphonema* spp. (including *G*. *cymbelliclinum* Reichardt & Lange-Bertalot, *G*. *decussis* (Ostrup) Lange-Bertalot & Metzeltin, *G*. *lateripunctatum* Reichardt & Lange-Bertalot, *G*. *micropus* Kützing, *G*. *olivaceum* var. *olivaceoides* (Hustedt) Lange-Bertalot, *G*. *parvulum* Kützing, *G*. *pumilum* var. *elegans* Reichardt & Lange-Bertalot, *G*. *pumilum* (Grunow) Reichardt & Lange-Bertalot, *G*. *truncatum* Ehrenberg, and *Gomphonema* sp.) and *Sellaphora* spp. (summing *S*. *seminulum* (Grunow) Mann and *S*. *stroemii* (Hustedt) Mann). Among them, 5 taxa had a significant relationship with the biomass of *D*. *geminata* (Fig 3). *Achnanthidium* spp. (mostly *A*. *minutissimum*), *Brachysira neoexilis* and the tube forming *Delicata delicatula* showed a positive relationship with said alga. The relative abundance of *Cocconeis placentula* and *Gomphonema* spp. (9 different taxa) significantly decreased in samples affected by *D*. *geminata* filaments (Fig 3). Diatom assemblage diversity, measured using the Shannon diversity index, was also negatively related to the *D*. *geminata* biomass (Fig 3).

**Fig 4:**
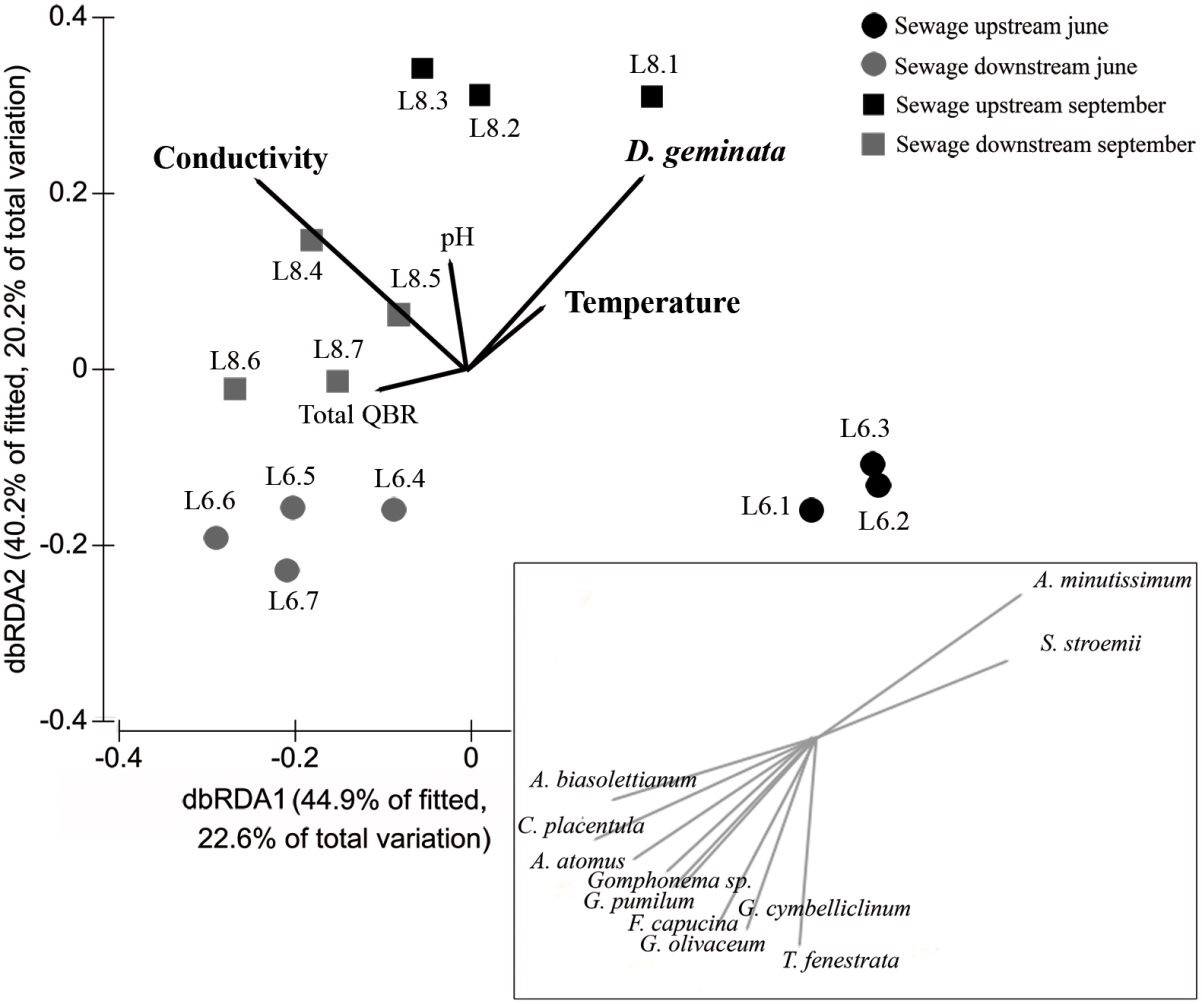
DISTLM analysis of diatom assemblage. Distance-based redundancy analysis (dbRDA) plot resulted from the DISTLM analysis that considered the diatom assemblage (species level) and the environmental variables at each site studied. The four groups of sites are represented by different symbols according to the sampling data and the site location along the longitudinal profile of the river. Variables included in the final model are shown, and those that are significant (p<0.05) are highlighted in black: *D*. *geminata* (filamentous mats density (g/m^2^)); Conductivity (μS/cm); Temperature (°C); pH; QBR (total value of this riparian quality index). Diatom species with a Spearman correlation coefficient of higher than 0.85 using the DISTLM analysis are shown in the lower right-hand corner of the figure.

Diatom assemblages varied between sites and on both dates according to the DISTLM analysis (Fig 4). *D*. *geminata* biomass was shown to be statistically significant in the DISTLM analysis, and the samples were plotted on the dbRDA graph according to the *D*. *geminata* biomass and sampling date. Diatom assemblages located downstream of the Lumbreras sewage discharge (sites L4, L5, L6 and L7) yielded similar results in both June and August, while the upstream diatom assemblages of sites L1, L2 and L3, all of which were affected by the massive growth, differ between dates due to the increase in *D*. *geminata* biomass as it grew. In Fig 4, we have shown on the dbRDA graph the species of diatoms that correlate strongly with the DISTLM analysis (those with a Spearman correlation coefficient higher than 0.85). *A*. *minutissimum* and *Sellaphora stroemii* showed a positive correlation with *D*. *geminata* levels, so their relative abundance increased in samples with large biomasses of filaments. Conversely, the ribbon forming *Fragilaria capucina* and several species of *Gomphonema* stand out by its contrary position to *D*. *geminata* on the dbRDA graph, since their relative abundance reduced as *D*. *geminata* biomass increased (Fig 4).

**Fig 5:**
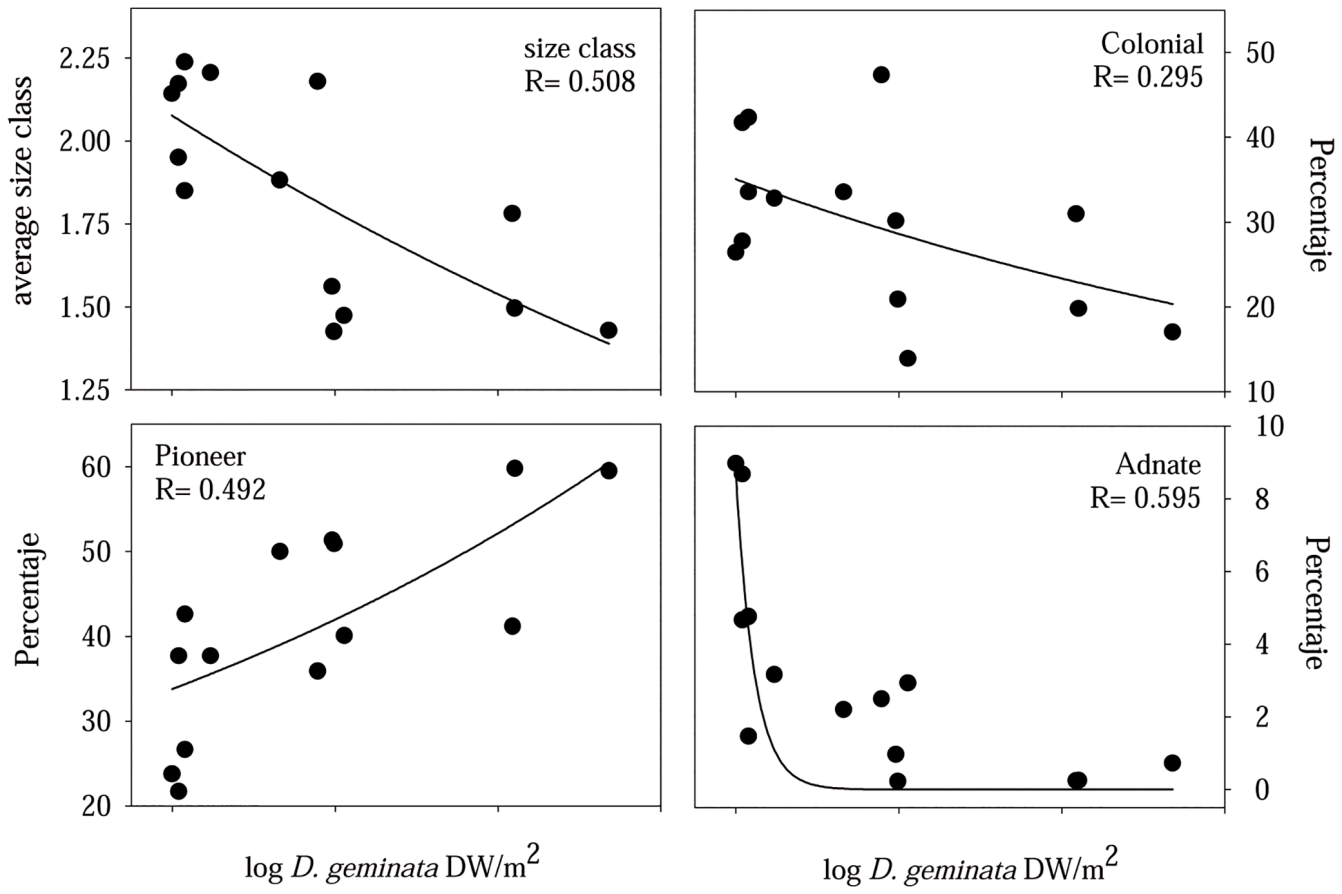
Relationship between *D*. *geminata* biomass and diatom assemblage traits. Exponential regressions between *D*. *geminata* biomass (log transformed) and macroinvertebrate assemblage trait categories that were shown to be statistically significant (p<0.05) related.

Regarding the functional structure of the diatom assemblage, four of the eight biological traits studied were shown to have a significant relationship to the biomass of the *D*. *geminata* (Fig 5). The relationship was positive for pioneer diatoms, while a higher biomass of filamentous mats was associated with a decrease in diatom size, and lower relative abundance levels of attached and colonial diatoms.

**Fig 6:**
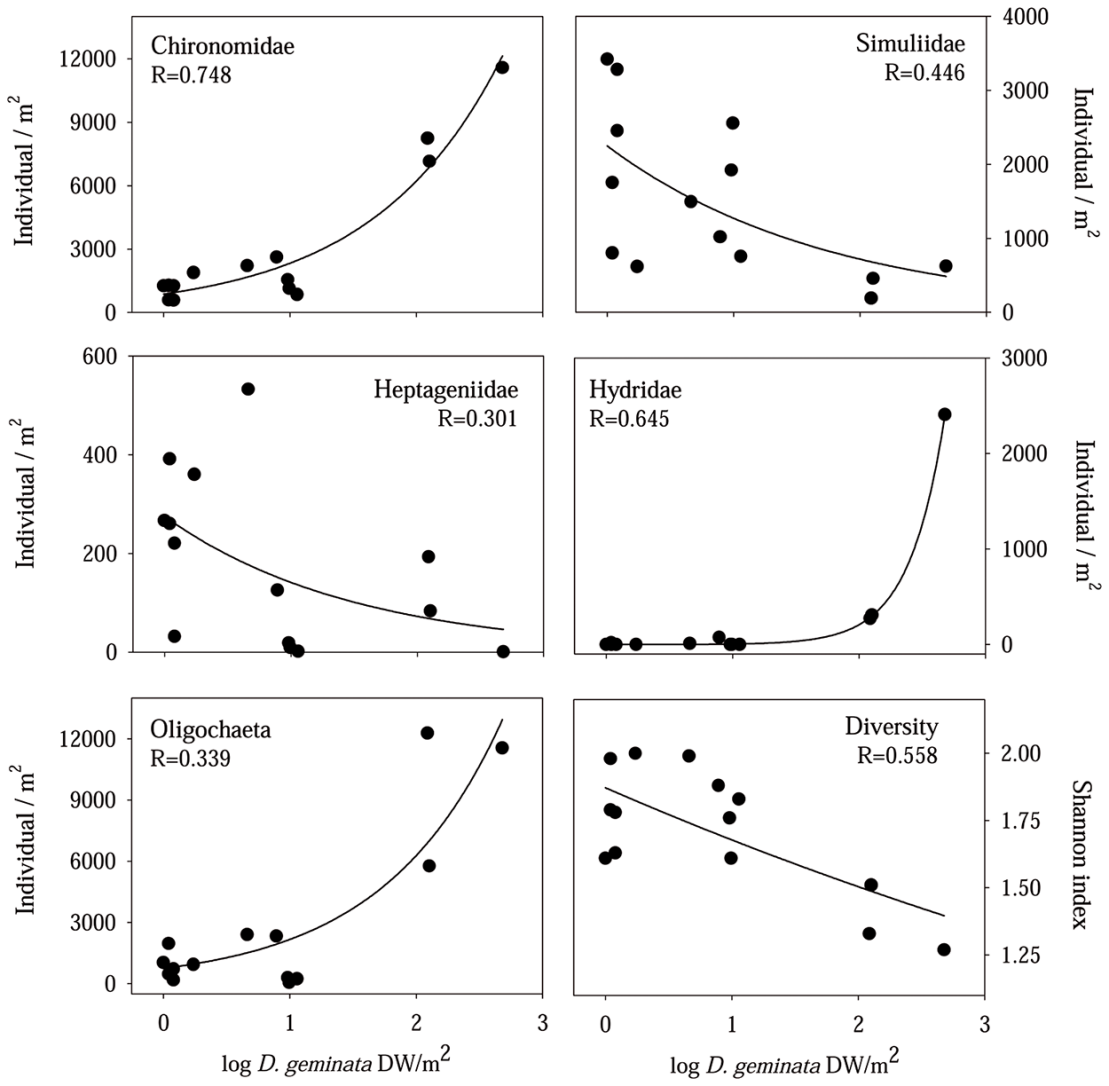
Relationship between *D*. *geminata* biomass and macroinvertebrate families and diversity. Exponential regressions between *D*. *geminata* biomass (log transformed) and macroinvertebrate taxa that were shown to be significant (p<0.05). (Exponential regressions between *D*. *geminata* biomass (log transformed) and the Shannon diversity index of the Macroinvertebrate assemblage at each site, which also resulted statistically significant (p<0.05) (lower right figure).

Regarding the taxonomic composition of the macroinvertebrate assemblage, 56 taxa were identified, generally to genus or sub-family level in Diptera. To achieve more consistent results in the regression analysis, different taxa were grouped to family level or higher, resulting in the identification of 39 taxa. The assemblage was clearly dominated by Baetidae (*Siphlonurus sp*. and mostly *Baetis sp*.), Ephemerellidae (*Serratella ignita*), Heptageniidae (*Ecdyonurus sp*., *Epeorus sp*. and *Rithrogena sp*.), Leuctridae (*Leuctra sp*.), Chironomidae (mostly Orthocladiinae, 97% of total chironomids), Simuliidae (Simuliini), Oligochaeta and Hydridae (*Hydra sp*.). All of these taxa showed a relative abundance of higher than 1% of the total macroinvertebrates when taking into account every sample, and altogether represented the 95% of the total number of macroinvertebrates. The five which were discovered to have a significant relationship with the *D*. *geminata* biomass are represented in Fig 6. Chironomidae, Oligochaeta and Hydra showed positive correlations; their density clearly increased in sites affected by the massive growth (Fig 6). In contrast, Heptageniidae and Simuliidae negatively correlated with *D*. *geminata* biomass, and were found to be less dense at sites that were most affected by the filamentous mats (Fig 6). Finally, the relationship between *D*. *geminata* biomass and the Shannon diversity index also showed a significant inverse relationship (Fig 6).

**Fig 7:**
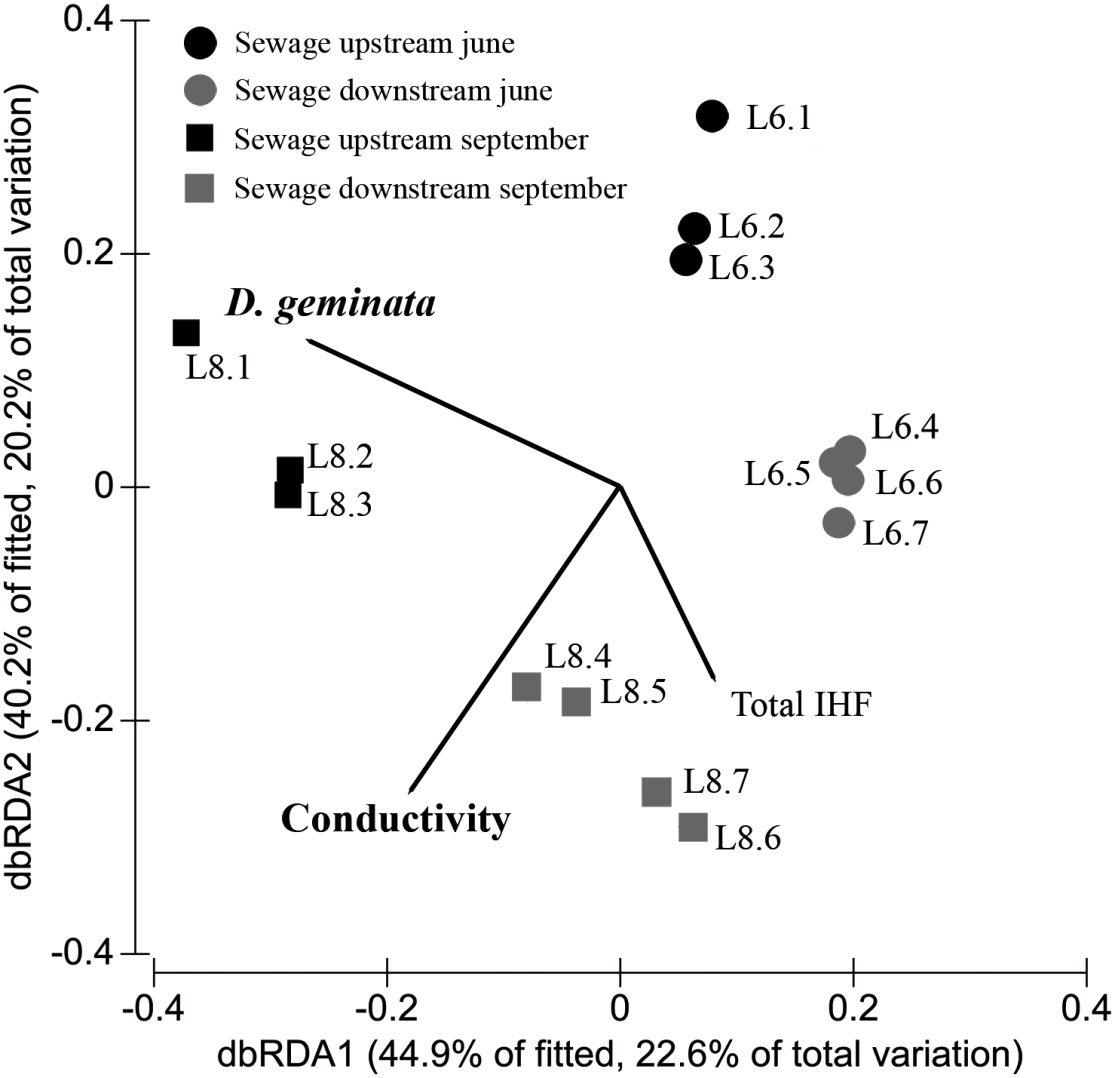
DISTLM analysis of the macroinvertebrate assemblage. Distance-based redundancy analysis (dbRDA) plot resulted from the DISTLM analysis, considering the macroinvertebrate assemblages (mostly genus or subfamily level) and the environmental variables studied at each site. The four groups of sites are represented using different symbols, according to the sampling data and the site location along the longitudinal profile of the river. Variables included in the final model are shown, and those that are significant (p<0.05) are highlighted in black: *D*. *geminata* (filamentous mats density (g/m^2^)); Conductivity (μS/cm); IHF (total value of this habitat quality index).

Macroinvertebrate assemblage showed an important variability among sites and dates, according to the DISTLM analysis (mostly based on genus or subfamilies densities) (Fig 7). The variables statistically significant in the DISTLM analysis were conductivity and *D*. *geminata* biomass. Accordingly, the sites were grouped on the dbRDA graph in line with how severe the *D*. *geminata* massive growth was (Fig 7).

**Fig 8:**
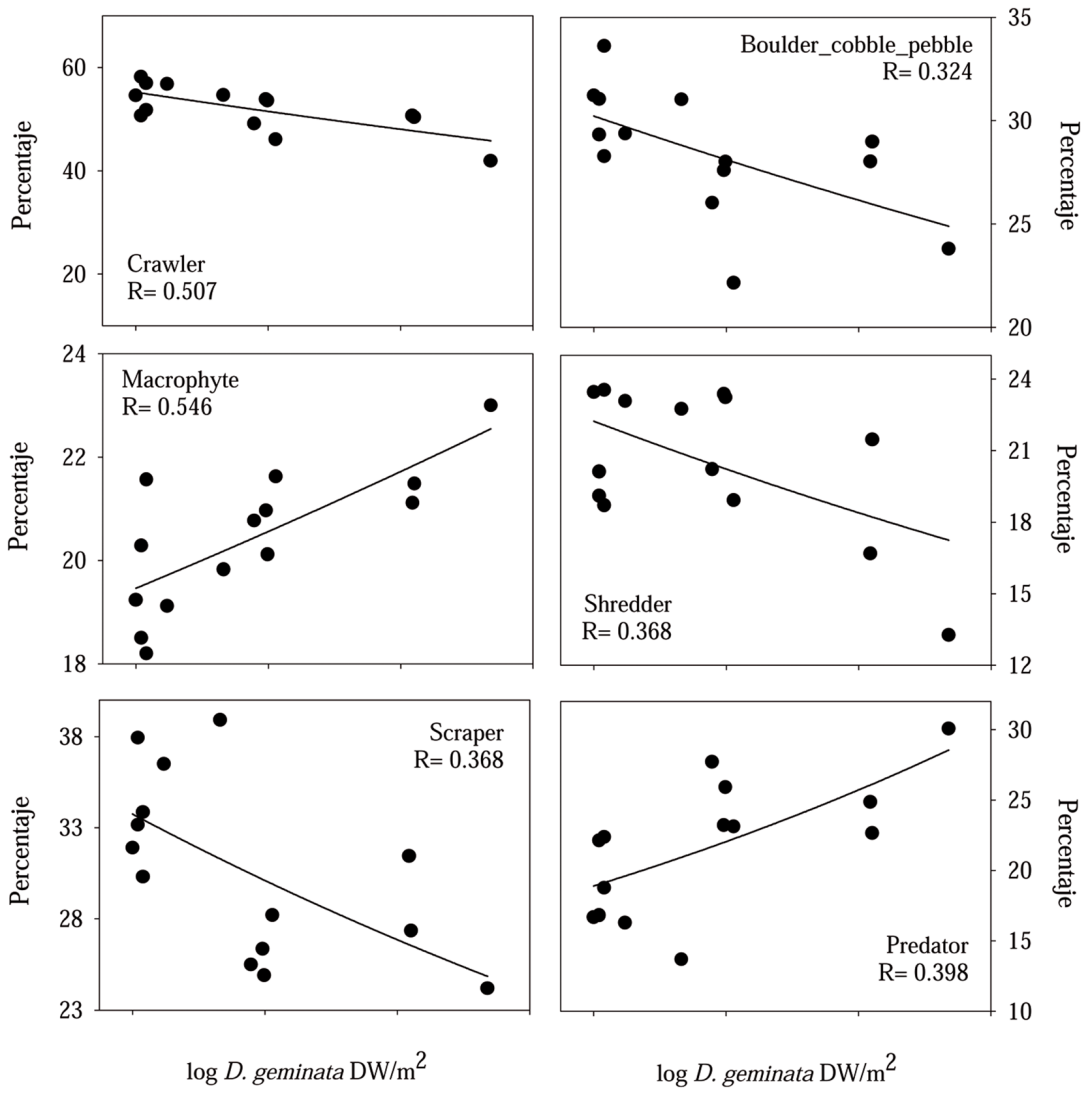
Relationship between *D*. *geminata* biomass and macroinvertebrate assemblage traits. Exponential regressions between *D*. *geminata* biomass (log transformed) and the six most abundant macroinvertebrate assemblage trait categories that were shown to be statistically significant (p<0.05) related.

Regarding the functional structure of the macroinvertebrate assemblage (mostly based on genus or subfamily level), and analyzed on the base of biological traits, 13 categories demonstrated a significant correlation with *D*. *geminata* biomass (only the six with the highest relative abundance are graphically presented -see Fig 8- since they can best explain the changes to the assemblage’s functional structure related *D*. *geminata*’s biomass). A higher density of filaments in the river substrate was associated with a decrease in the percentage of crawlers, shredders, scrapers and taxa adapted to live in the coarse substrate, as boulders, cobbles and pebbles (Fig 8). The number of predators and taxa adapted to live on macrophytes correlated positively with an increase in *D*. *geminata* biomass (Fig 8).

## Discussion

The main environmental variable related to macroinvertebrate and diatom assemblages variation among sites resulted *D*. *geminata* biomass. Development of *D*. *geminata* massive growth is associated to other studied factors, especially SRP and QBR and IHF indexes, as we profoundly discusses in Ladrera *et al*. [10]. The remaining studied variables did not play an important role for river community alteration, since every studied sites showed similar and high dissolved oxygen concentration, low temperature and conductivity and slightly alkaline waters, according to other rivers affected by *D*. *geminata* (e.g. Bhatt *et al*.[48]).

**Fig 9:**
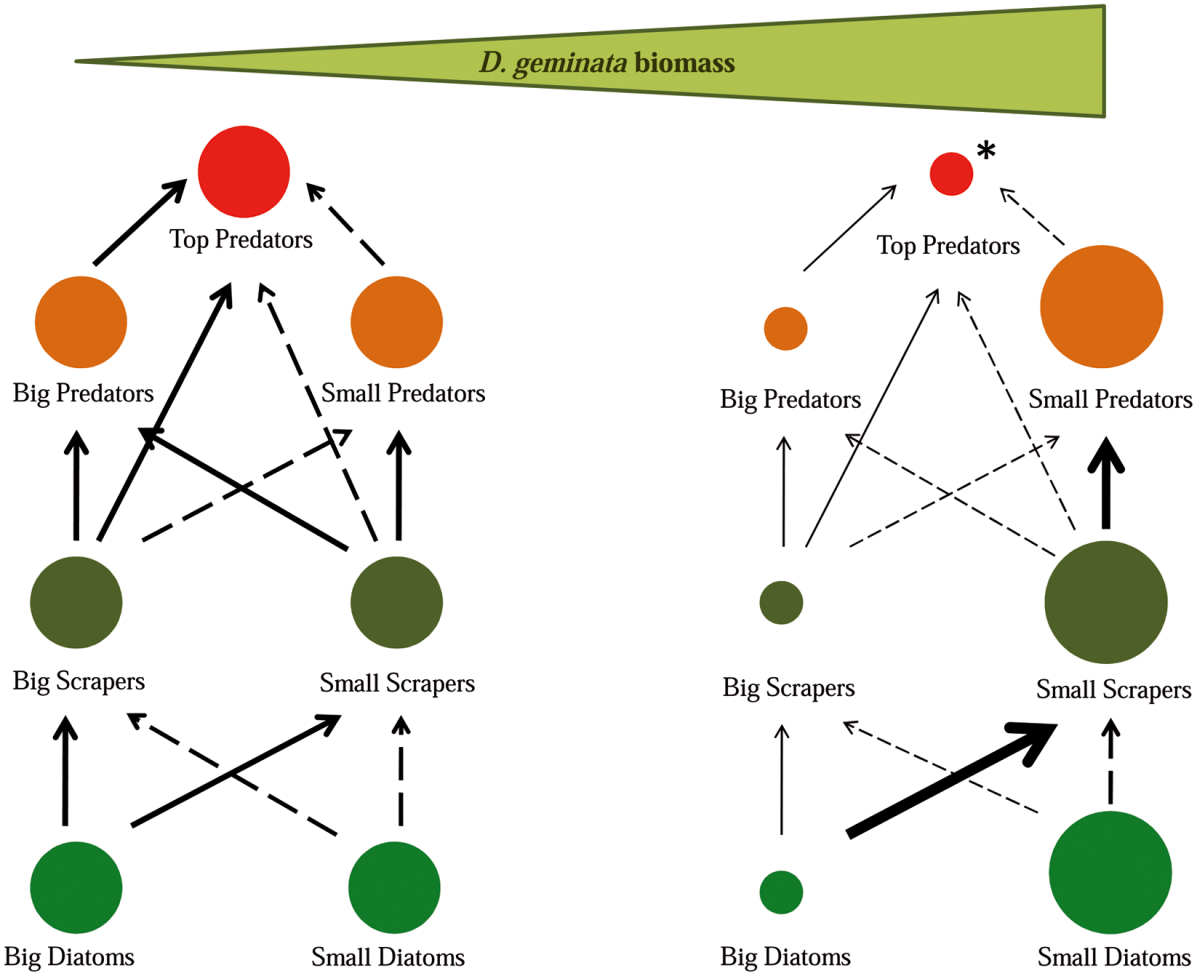
Trophic community alterations. Diagram of the trophic interactions related to increased *D*. *geminata* biomass levels in the river according to our results and based on Rodriguez-Lozano *et al*.’s [18] diagram methodology. Circumference size represents the density of each kind of organism. The arrows represent the intensity of trophic interactions, increasing from the thinner dotted arrow to the thicker continuous one. The main organisms included in each category are: Small diatoms (A. *minutissimum*, *B*. *neoexilis*); Big diatoms (*C*. *placentula*, *Gomphonema sp*.); Small scrapers (Orthocladiinae); Big scrapers (Heptageniidae); Small predators (Hydra); Big predators (Perlidae); Top predator (*Salmo trutta*). *Top predator level has not been studied in the present work, but we are hypothesizing based on existing literature and considering its important role in the food web.

Massive *D*. *geminata* growth profoundly altered the river community both directly and indirectly, causing food web alterations as schematically represented in Fig 9. The increase in *D*. *geminata* biomass led to clear taxonomical and functional changes to the macroinvertebrate assemblage, and this took place in several ways. The increased biomass of the *D*. *geminata* filaments brought about a progressive decrease in the number of crawlers, shredders, scrapers and invertebrate taxa adapted to live on boulders, cobbles and pebbles. These trait categories were impacted considerably since filamentous mats completely cover the hard substrate, which makes it difficult for taxa that move and/or feed on it to survive. This finding is in accordance with our first hypothesis. Among these taxa, and taking into account the physical structure of the filamentous mats, those of greater size were especially affected, as has been seen in other studies [16,49]. Thus, in river sections with massive *D*. *geminata* growths, the dense filament framework hampers the movement of large scrapers, such as Heptageniidae, on the substrate.

Other taxa significantly affected by a high biomass of filaments, as we hypothesized, are those that live fixed to the substrate, such as Simuliidae, as there is a significant reduction in the area of substrate surface where they can fix themselves [22,50].

In spite of this, the newly formed filamentous framework favors smaller opportunistic taxa that are capable of adapting to the new environmental conditions such as Chironomidae and Oligochaeta, which also benefit from less competition for food, a consequence of the general decrease in the numbers of the organisms mentioned previously, and are further favored by the increase in the amount of FPOM (Fine Particulate Organic Matter) accumulated between the filaments due to reduced water velocity.

Aside from chironomids and Oligochaeta, the new environmental conditions created by *D*. *geminata* particularly favored Hydra, which can easily attach themselves to macrophyte structures [51]. Moreover, their small size compared to other predators [44] allows Hydra to better adapt to filamentous environments, and they benefits further from the increase in oligochaete and chironomid numbers, upon which they prey. Nevertheless, Hydra are not as efficient in the control of their populations as large predators are. The large predator Perlidae, a common family in the Lumbreras River, was completely absent at site L1, where the filamentous mats reached the highest densities. As happens with large crawlers and scrapers, the mesh of filaments impedes the movement of large predators and makes the capture of prey more difficult, a further indirect effect that favors small macroinvertebrates.

Although we have not studied the fish assemblage, the reduction of invertebrate size in the community could have negative implications for fish bioenergetics [52], in line with the observations of Jellyman and Harding [16], the first recent work to study how massive *D*. *geminata* growths can have detrimental effects for fish. These authors [16] showed that brown trout (*Salmo trutta*), the main fish in our studied area, can be affected by the new environmental conditions in *D*. *geminata* impacted sites, reducing their ability to capture prey, increasing their predatory risk and providing no suitable spawning areas, beyond the indirect effects mediated by the macroinvertebrate assemblage alteration. As a result, taking into account the top-down control, we hypothesize that the increase of Hydridae and chironomids in sites affected by massive *D*. *geminata* growth observed in the present study could also be a consequence of the “messopredator release” and “prey release” regulations [18]. It would occur after a reduction in the numbers of trout and larger invertebrate predators, according to what has been observed in Mediterranean rivers under other kinds of ecosystemic pressures [18]. We wish to emphasise the need for studies aimed at understanding the effects of *D*. *geminata* on the fish assemblage together with the macroinvertebrate and diatom assemblages.

The significant increase in chironomid numbers, mainly Orthocladiinae, related to the decrease in numbers of large scrapers and predators in sites affected by filamentous mats of *D*. *geminata* confirms our second hypothesis, and demonstrates indirect effects upon the diatom assemblage. Different studies have shown that chironomids feed primarily, along with FPOM, on large diatoms [53–55], since they cannot capture diatoms of a smaller size with the same efficiency. In the present study, Orthocladiinae density, mainly *Cricotopus spp*. and *Eukieferiella spp*., increased over ten times in samples affected by the massive growth, reaching values of higher than 10000 ind./m^2^. This high Chironomidae density in sites affected by massive *D*. *geminata* growth, has also been determined by other authors [11,16,21,49], and lead to strong grazing pressure upon larger diatoms, thus contributing to the increased dominance of small diatoms.

Moreover, small and pioneer diatoms result also favored by the newly created microenvironment of filamentous mats to which they can attach themselves [56,57]. The pioneering nature and attaching ability of the small diatom *A*. *minutissimum* allow it to be the first to colonize *D*. *geminata* filaments, and thanks to its rapid instantaneous growth, its population was found to rise significantly at sites affected by the massive growths. Likewise, the dominance of *A*. *minutissimum* in *D*. *geminata* presence has also been frequently cited in other geographical areas [11,23,24,58–61]. We determined a positive relationship between *D*. *geminata* biomass and the relative abundance of other small diatoms such as *S*. *stroemii*.

Simultaneously, *D*. *geminata* exerted pressure by direct competition on large attached species, be they stalked or adnate, in accordance with our first hypothesis. The displacement of species with similar ecological requirements to *D*. *geminata*, such as *C*. *placentula*, *Gomphonema* spp. and *Fragilaria* spp, most likely resulted from the higher growth rates and the total substrate occupation of this invasive algae [59,62].

The community alterations resulting from massive *D*. *geminata* growth leads to a significant reduction in diatom and macroinvertebrate diversity, in contrast with the findings of Gillis and Lavoie [23] and Sanmiguel *et al*. [24], which described an increase in diatom divertity at sites affected by massive *D*. *geminata* growth. These authors associated the increase in diversity with the new microhabitat created by the *D*. *geminata* filaments. We disagree with their findings, as we believe that two factors led to a reduction in diatom assemblage diversity: many larger attached species were found to be negatively affected by ecological interactions with the massive growths, whilst the increase in new substrate and chironomid numbers favors certain small fast-growing pioneer species and allows them to become largely dominant. These results, together with the reduction in macroinvertebrate diversity highlighted in the present work and in other studies [22,24] show a simplification in the river community structure following massive *D*. *geminata* growth.

According to our third hypothesis, the incidence of river community composition and function increasingly correlates with *D*. *geminata* biomass, contrary to what is proposed by some other authors [23], who consider that beyond a certain *D*. *geminata* accrual, further increases in biomass do not have an impact on community structure. In the present study, communities in sites affected by the massive growth continue to exhibit change either in terms of taxa composition, or functionality, as the filamentous mat density continues to increase over time.

These results allow us to consider *D*. *geminata* an ecosystem engineer, since it affects both the stream community and the food web by physically modifying both habitats and resources [12,13]. In light of this, *D*. *geminata* joins the group of invasive species that affect the hosting ecosystem via habitat engineering alteration [15,63]. The frame of massive filaments significantly alters not only the specific composition, but also the functional structure of the diatom and macroinvertebrates, which leads to a complete alteration in how the food web functions. This occurs either via direct interactions (exclusive competition or new substrate provision for diatoms, the displacing of bigger species for macroinvertebrates) as well as via indirect interactions (decrease in number of big predators scales down the food web to further favour certain primary producers -smaller diatom species-). The resulting system is made up of smaller organisms that concentrate major part of the biomass, and features a simplified food web.

## Conclusions

This work provides further evidence of the specific *D*. *geminata* effects upon the resident community ecology. It considered taxonomic composition, functional structure and trophic relations, in order to produce thorough results. It is the first study that jointly considers the taxonomic composition and the functional structure of diatom and macroinvertebrate assemblages, and how the alga interferes with trophic relations, to study the river’s biotic functioning alterations that are associated with *D*. *geminata*.

Massive growth of this invasive alga causes diversity reduction and considerable alterations to both communities, owing to the biomass of its filamentous mats. Community alterations are associated with the new environmental conditions, which cause a biological top-down control in the aquatic ecosystem composition and structure. *D*. *geminata* mats hamper the survival of large invertebrates and predators since they are not capable of moving and feeding on the substrate once colonized by the filaments. The reduced risk of predation from larger organisms, and diminished competition for food from big scrapers favors smaller organisms, mainly chironomids, which can move freely inside the filamentous mats. Consequently, chironomids exercise a strong grazing pressure on larger diatoms, which are already in direct competition with *D*. *geminata*, and contribute to the dominance of small fast-growing diatoms, which pioneer the brand new filamentous environment, where they can fix themselves and live free of competition.
